# The use of machine learning in rare diseases: a scoping review

**DOI:** 10.1186/s13023-020-01424-6

**Published:** 2020-06-09

**Authors:** Julia Schaefer, Moritz Lehne, Josef Schepers, Fabian Prasser, Sylvia Thun

**Affiliations:** 1 Technische Universität Berlin, Berlin, Germany; 2 Berlin Institute of Health (BIH), Berlin, Germany; 3 Charité – Universitätsmedizin Berlin, Berlin, Germany; 4 Hochschule Niederrhein – University of Applied Sciences, Krefeld, Germany

**Keywords:** Rare diseases, Machine learning, Scoping review

## Abstract

**Background:**

Emerging machine learning technologies are beginning to transform medicine and healthcare and could also improve the diagnosis and treatment of rare diseases. Currently, there are no systematic reviews that investigate, from a general perspective, how machine learning is used in a rare disease context. This scoping review aims to address this gap and explores the use of machine learning in rare diseases, investigating, for example, in which rare diseases machine learning is applied, which types of algorithms and input data are used or which medical applications (e.g., diagnosis, prognosis or treatment) are studied.

**Methods:**

Using a complex search string including generic search terms and 381 individual disease names, studies from the past 10 years (2010–2019) that applied machine learning in a rare disease context were identified on PubMed. To systematically map the research activity, eligible studies were categorized along different dimensions (e.g., rare disease group, type of algorithm, input data), and the number of studies within these categories was analyzed.

**Results:**

Two hundred eleven studies from 32 countries investigating 74 different rare diseases were identified. Diseases with a higher prevalence appeared more often in the studies than diseases with a lower prevalence. Moreover, some rare disease groups were investigated more frequently than to be expected (e.g., rare neurologic diseases and rare systemic or rheumatologic diseases), others less frequently (e.g., rare inborn errors of metabolism and rare skin diseases). Ensemble methods (36.0%), support vector machines (32.2%) and artificial neural networks (31.8%) were the algorithms most commonly applied in the studies. Only a small proportion of studies evaluated their algorithms on an external data set (11.8%) or against a human expert (2.4%). As input data, images (32.2%), demographic data (27.0%) and “omics” data (26.5%) were used most frequently. Most studies used machine learning for diagnosis (40.8%) or prognosis (38.4%) whereas studies aiming to improve treatment were relatively scarce (4.7%). Patient numbers in the studies were small, typically ranging from 20 to 99 (35.5%).

**Conclusion:**

Our review provides an overview of the use of machine learning in rare diseases. Mapping the current research activity, it can guide future work and help to facilitate the successful application of machine learning in rare diseases.

## Background

Diseases that affect fewer than 5 patients per 10,000 are defined as rare in Europe [1]. But rare diseases are only rare when considered individually. With more than 6000 known rare diseases [2], their collective global health burden is high, and recent estimates report a population prevalence of at least 3.5–5.9% [3]. (The true prevalence is probably higher, as for many rare diseases epidemiological data are scarce.) Moreover, due to their often genetic origin and early disease onset – often in infancy or childhood – most rare diseases follow patients for large parts of their lives, thus further exacerbating the disease burden.

More than 80% of rare diseases affect fewer than one patient in a million [3]. This means that, for most rare diseases, even experienced physicians with a lot of patient contact never see a single patient in their lifetime. Correctly diagnosing patients is therefore difficult: According to a survey from 2013, it takes, on average, more than 5 years, eight physicians and two to three misdiagnoses until a rare disease patient receives the correct diagnosis [4]. Once correctly diagnosed, the challenges continue: Due to the small patient numbers, commercial incentives for developing medications are often low (although policies and legislations aim to raise financial incentives for developing rare disease treatments). Furthermore, the pathophysiological mechanisms underlying rare diseases are often not well understood. As a consequence, many rare diseases lack adequate treatment options. Improving the diagnosis and treatment of rare diseases is therefore an important public health concern.

One valuable approach for improving medical care for rare disease patients are initiatives and networks that aim to bundle data and expertise about rare diseases so that healthcare providers can easily access and exchange relevant information. One of the most extensive knowledge bases for rare diseases is Orphanet [5], which provides information about, for example, disease epidemiology, associated genes, inheritance types, disease onsets or references to terminologies, as well as links to expert centers, patient organizations and other resources. Other European initiatives include RD-Connect, which combines registries, biobanks and genetic data with bioinformatics tools to provide a central resource for research on rare diseases [6]; the European Reference Networks (ERNs), which provide an IT infrastructure that allows healthcare professionals to collaborate on virtual panels to exchange knowledge and decide on optimal treatments [7]; and the European Joint Programme on Rare Diseases (EJP RD), a multinational cooperation aiming to create an ecosystem that facilitates research, care and medical innovation in the field of rare diseases [8]. In the US, the Undiagnosed Diseases Network (UDN) brings together experts to diagnose and treat patients with rare conditions [9]. And in Germany, a new national initiative, the Collaboration on Rare Diseases (CORD-MI), aims to improve the documentation and data exchange of rare diseases across German university hospitals [10].

In addition to these collaborative efforts and international platforms, another important factor that can improve the situation for rare disease patients are advances in information technology – particularly in the field of artificial intelligence (AI) and machine learning. AI and machine learning typically use large, multivariate datasets to “train” algorithms, which are then used to make predictions on new data (for example, by classifying tumors in radiological images as benign or malignant). Importantly, the computations by which these methods generate their output are not explicitly coded by a programmer, but instead are implicitly “learned” by the algorithm from example data (hence the term “machine learning”). AI and machine learning are increasingly applied in medicine and healthcare [11, 12] and, in some areas, are beginning to achieve (and sometimes even surpass) human-level performance [13–15]. Given the specific challenges in diagnosis and treatment discussed above, rare diseases can particularly benefit from AI and machine learning technologies: While it is virtually impossible for a physician to memorize information about thousands of rare diseases, modern computers can easily “memorize” huge quantities of digital information. If the computer can also extract and use this information in a meaningful way – for example, by classifying patients into disease groups or predicting outcomes – this has a high potential for improving diagnosis and treatment. Previous research, for example, has shown that an AI expert system that calculates disease probabilities based on patient symptoms can potentially accelerate rare disease diagnoses [16]. Using methods of computer vision and deep learning, another system, Face2Gene, can assist physicians in diagnosing rare genetic conditions based on photographs of patients’ faces [17].

Despite its potential for improving the quality of care for patients, the use of machine learning in the field of rare diseases has not been comprehensively reviewed (but see [18] for an overview with a special focus on congenital disorders of glycosylation). For example, it is unclear in which rare diseases machine learning is applied, which algorithms are typically used, which medical applications are studied (e.g., diagnosis, prognosis or treatment) and which type of input data is used. In this scoping review, we explore the scientific literature to answer these questions and investigate how machine learning is currently used in the context of rare diseases. Providing an overview of research in machine learning and rare diseases, our review can help to direct future work in this area, for example, by pointing to gaps in research or to promising fields for future study.

## Methods

We opted to perform a scoping review because this type of review is best suited to map research activity in a broad and heterogeneous field such as machine learning and rare diseases (unlike typical systematic literature reviews that focus on more specific research questions) [19–22]. Where applicable, we follow the guidelines of the PRISMA extension for scoping reviews (PRISMA-ScR) [23]. No review protocol was registered for this study.

To identify scientific articles that apply machine learning in the field of rare diseases, we systematically searched the literature on PubMed. The search string was constructed by concatenating general terms related to machine learning (“machine learning”, “artificial intelligence”) and rare diseases (“rare disease”, “orphan disease”), as well as names and synonyms of 381 specific rare diseases. These specific diseases comprised all rare diseases listed by Orphanet [5] with a point prevalence of 1–5 per 10,000 (146 diseases) or 1–9 per 100,000 patients (235 diseases). For many of these diseases, Orphanet provides PubMed search strings that were used to construct the search (for example, “Deletion[ti] 4p[ti] OR 4p syndrome[tw] OR wolf hirschhorn[tw] OR (chromosome deletion[mh] chromosome 4[mh])” for the Wolf-Hirschhorn syndrome). For diseases where no such search strings were available from Orphanet, the disorder name was used (for the exact search terms, see Additional file 1). The search was first conducted on January 2, 2020. During the revision process of the manuscript the search term was slightly modified and the search was conducted again on May 5, 2020. (The initial search term used in January had included some specific machine learning methods, such as “neural network” and “deep learning”, which could have biased the search results towards these methods. These search terms were omitted in the final search.)

To be included in this review, the studies identified in the search had to fulfill the following eligibility criteria: rare disease topic; use of at least one machine learning method (and a description of the machine learning algorithm in sufficient detail to extract the basic information analyzed in this review); publication date between January 1, 2010, and December 31, 2019; publication as original research in a peer-reviewed journal or conference proceeding (i.e., review articles were excluded); publication in English or German; application of machine learning to human patient data or scientific texts or literature (i.e., articles using animal or simulation data were excluded). As our review does not aim to answer a specific clinical question, but instead explores the use of machine learning in rare diseases from a general perspective, we did not restrict eligibility to specific patient populations, interventions (except, of course, the use of machine learning), control groups or outcomes. For the same reason, we did not assess bias in the studies.

After having selected relevant studies according to the eligibility criteria, the following data were extracted from the articles: 1) rare disease (diseases were specified using the Orphanet disorder name; studies investigating more than one disease were categorized as “Diverse”); 2) rare disease group (according to the “preferential parent” of the disease as defined in the hierarchy of the Orphanet classification [24], e.g., rare neurologic disease, rare hematologic disease etc.); 3) prevalence of rare disease (according to epidemiological information from Orphanet); 4) year of publication; 5) country where study was conducted (according to the senior author’s affiliation); 6) number of patients (if applicable / available); 7) medical application (i.e., “Diagnosis”, “Treatment”, “Prognosis” or “Basic research”); 8) type of input data; 9) type of algorithm; 10) validation of algorithm on external data or against human expert.

For the variables “medical application”, “type of input data” and “type of algorithm”, categories were defined into which the studies were grouped. Categories were defined in a two-step process: First, the medical application, input data and machine learning algorithm were assessed in detail for each study (for example, a study might be described as aiming to distinguish patients from healthy controls, using a convolutional neural network on magnetic resonance imaging data of the brain). Based on these detailed data, two of the authors (JuS and ML) then defined meaningful, more general categories into which studies were grouped (for the previous example, this would be “Diagnosis” as medical application, “Images” as input data and “Artificial neural network” as type of algorithm). We did not rely on typical textbook categorizations of these variables (for example, classifying machine learning algorithms into supervised, unsupervised or reinforcement learning), as these categorizations were found not to be sufficiently informative and did not adequately reflect the studies (reinforcement learning, for example, does not play a significant role in the context of rare diseases). Instead, we defined a set of categories that aimed for a balance between sufficient detail and meaningful generalizations. This resulted in roughly ten categories for “type of input data” and “type of algorithm”. Note that a study could be grouped into more than one category when it used more than one type of input data or algorithm. Table 1 shows the variables extracted from the studies and the categories used for each variable.

**Table 1:** Data extracted from the studies

Variable	Categories	Definition	Example(s)
**Rare disease**	All rare diseases described at least once in the studies (studies investigating more than one rare disease were categorized as “Diverse”)	Orphanet disorder name	Cystic fibrosis, Sickle cell anemia, Gaucher disease
**Disease group**	All disease groups of the 381 specific diseases included in the search as well as disease groups of other diseases identified in the studies	Orphanet disease group as defined by the preferential parent in the classification hierarchy	Rare neurologic disease, Rare respiratory disease, Rare endocrine disease
**Publication year**	Years from 2010 to 2019	Year of the publication date of the article	
**Country of study**	All countries that published at least one article	Country of institution of senior (i.e. last) author of the study	
**Medical application**	Diagnosis	Studies aiming to correctly diagnose patients	Classification of cases and controls or different disease subtypes, Identification of biomarkers, Deep phenotyping, Decision support
	Treatment	Studies aiming to improve treatment or develop new therapies	Detection of therapeutic targets, Identification of binding proteins
	Prognosis	Prediction of a patient-relevant endpoint	Prediction of complication, disease onset, survival, disease progression, Risk estimation
	Basic research	Other basic research not classified into one of the categories above	Exploration of molecular disease mechanisms
**Patient number**	“< 20”, “20–99”, “100–1000”, “> 1000”, “not applicable / no information”	Number of patients included in the study	
**Input data**^**a**^	Clinical test score	Data from a clinical test score	Glasgow Coma Scale, ALS Functional Rating Scale
	Demographic data	General patient characteristics	Age, Sex, Ethnicity
	Functional test data	Data from physiological tests	ECG, EEG, EMG, gait pattern, pulse, blood pressure, eye movements
	Images	Data from medical imaging	MRI, PET, CT, retinal images, face photographs
	Laboratory data	Data from laboratory test	Blood glucose, platelet counts, creatinine
	Literature	Data extracted from scientific texts	Published literature, NCBI disease corpus
	Medication data	Data about medication	Use of antibiotics, medication plan
	Omics data	Molecular data	Genomics, Proteomics, Metabolomics, Epigenomics
	Patient / Family history	Data from patients’ or relatives’ past medical history	Pre-existing conditions, parental data
	Other EHR data	Other data from electronic health records	Diagnoses, procedures, other medical records
	Other	Other types of input data	Questionnaire or interview data, donors’ characteristics in HSCT
**Type of algorithm**^**a**^	Artificial Neural Network		Convolutional neural network, Recurrent neural network, Multi-layer perceptron
	Bayesian Methods		Naïve Bayes
	Clustering		k-means clustering, Hierarchical clustering
	Decision Tree		Decision tree
	Discriminant Analysis		Linear discriminant analysis
	Ensemble Methods		AdaBoost, Random forest
	Instance-based Learning		k-nearest neighbor
	Regression (logistic)		Logistic regression
	Regression (other)		Linear regression
	Support Vector Machine		Support vector machine
	Other	Algorithms not classified into one of the categories above	Reinforcement learning, Graphical models
**External validation**	yes / no	Performance of algorithm tested on external data or against a human expert	Comparing automated scoring of chest radiographs with scoring by radiologists

^a^For these variables, a study could be assigned to more than one category

Study selection and data extraction were performed by the first author (JuS). For unclear cases, the selection and data extraction were reviewed by the second author (ML) and discussed until a consensus was reached. Extracted data were saved in a spreadsheet for subsequent analysis.

To get an overview of the use of machine learning in rare diseases, we then explored, for each of the variables described above, how many studies were in each category. We also explored possible gaps in research by comparing the distribution of rare disease groups investigated in the studies with the “baseline” distribution of disease groups of the 381 diseases included in our search. For this, we calculated the percentage of diseases within each disease group for the diseases from the studies as well as for the diseases from the search list and then calculated their difference (in percentage points). The magnitude of the difference then indicated which disease groups were underrepresented (or overrepresented) in the studies. All data analyses and visualizations were done with R [25] and the tidyverse packages [26].

## Results

**Fig. 1:**
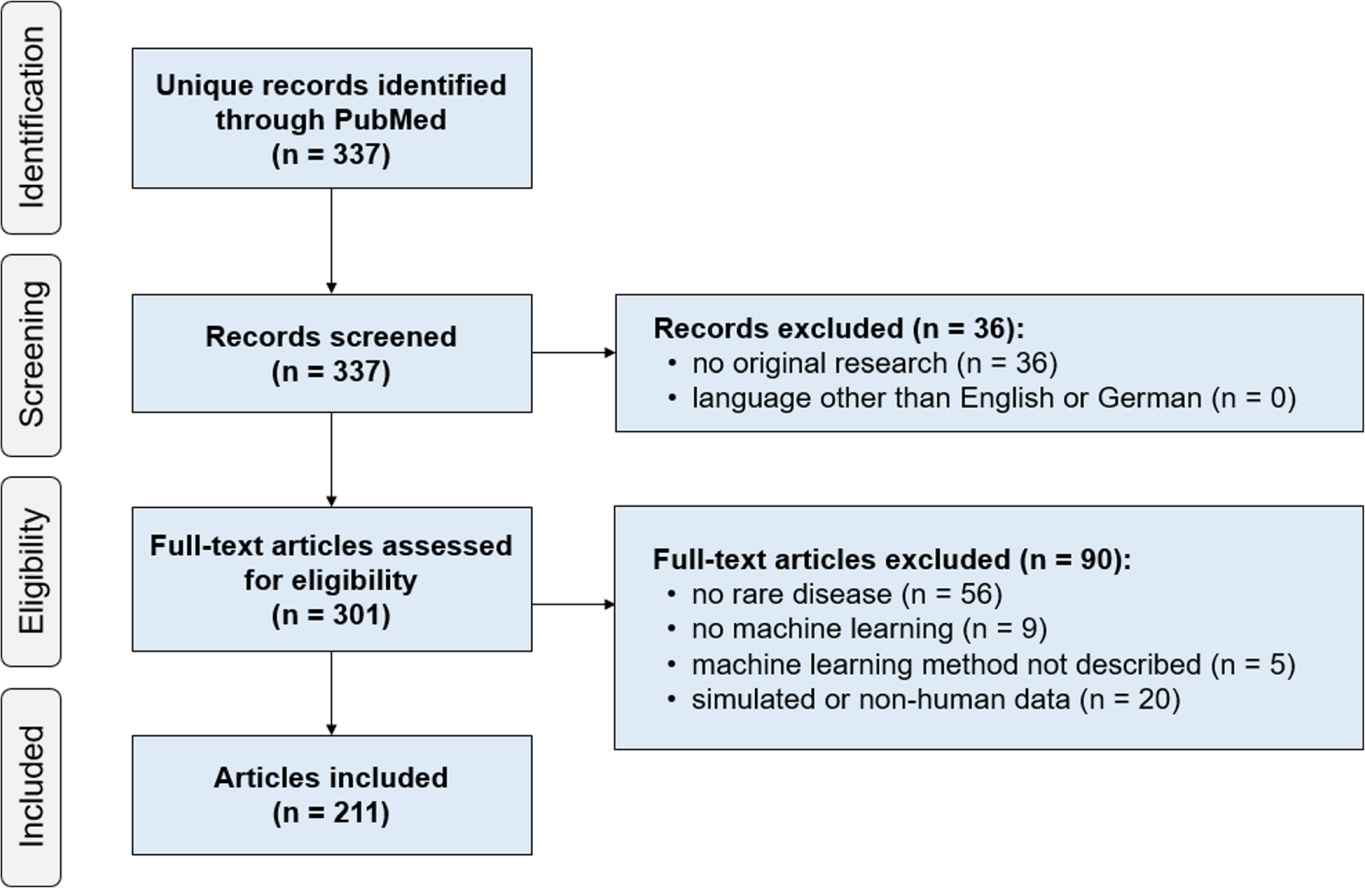
Selection of sources of evidence

The literature search identified a total of 337 unique records. After screening and assessing the articles for eligibility, 211 articles were included in the final analysis (Fig. 1; the list of articles and extracted data is included in Additional file 1). Though not a strict inclusion criterion, all articles in the final selection were in English (no German-language articles were eligible for inclusion).

**Fig. 2:**
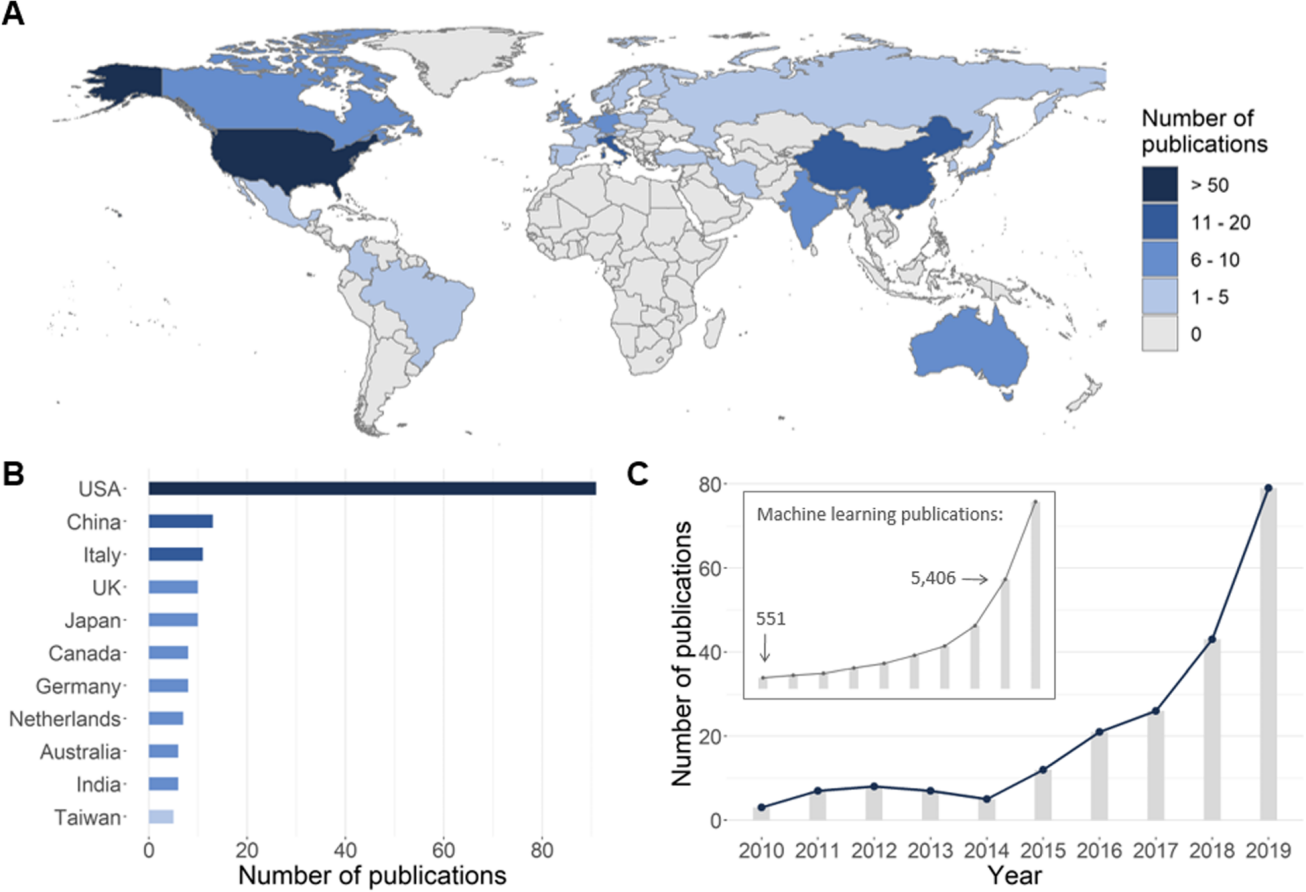
World map showing publications by country (**a**); countries with more than five publications (**b**); total number of publications per year (**c**; for comparison, the inset shows the publication trend for machine learning in general)

The studies originated from 32 different countries, with the largest number of publications (*n* = 91, 43.1%) coming from the United States (Fig. 2a, b). Over the 10-year time period considered in this review, publication numbers increased from 3 publications in 2010 to 79 publications in 2019. This increase in publication numbers appeared to parallel the increase of publications about machine learning in general (Fig. 2c).

Seventy-four different rare diseases were investigated in the studies. Of these 74 diseases, 71 were part of the list of the 381 rare diseases that were explicitly included in the search string (18.6%). Three diseases not explicitly listed in the search string – multiple osteochondromas, Fanconi anemia, juvenile idiopathic arthritis – were additionally described in the studies (these studies were identified via the generic search terms “rare disease” or “orphan disease”). Of the 74 diseases, 41 (55.4%) had a prevalence of 1–5 / 10,000 patients, 31 (41.9%) had a prevalence of 1–9 / 100,000, and 2 (2.8%) had a prevalence of 1–9 / 1000,000. The diseases most frequently investigated in the studies were amyotrophic lateral sclerosis, systemic lupus erythematosus, moderate and severe traumatic brain injury and cystic fibrosis (Table 2; note that some studies investigated more than one disease).

**Table 2:** Rare diseases most frequently investigated in the studies (all diseases appearing in five or more studies are listed)

Rare disease	Orpha number	Prevalence	Number of studies
Amyotrophic lateral sclerosis	803	1–9 / 100,000	16 (7.6%)
Systemic lupus erythematosus	536	1–5 / 10,000	14 (6.6%)
Moderate and severe traumatic brain injury	90056	1–5 / 10,000	12 (5.7%)
Cystic fibrosis	586	1–9 / 100,000	10 (4.7%)
*More than one rare disease investigated*	*–*	*–*	*10 (4.7%)*
Huntington disease	399	1–9 / 100,000	9 (4.3%)
Down syndrome	870	1–5 / 10,000	7 (3.3%)
Preeclampsia	275555	1–5 / 10,000	7 (3.3%)
Acquired aneurysmal subarachnoid hemorrhage	90065	1–5 / 10,000	6 (2.8%)
Systemic sclerosis	90291	1–5 / 10,000	6 (2.8%)
Fragile X syndrome	908	1–5 / 10,000	5 (2.4%)
Retinopathy of prematurity	90050	1–5 / 10,000	5 (2.4%)

**Fig. 3:**
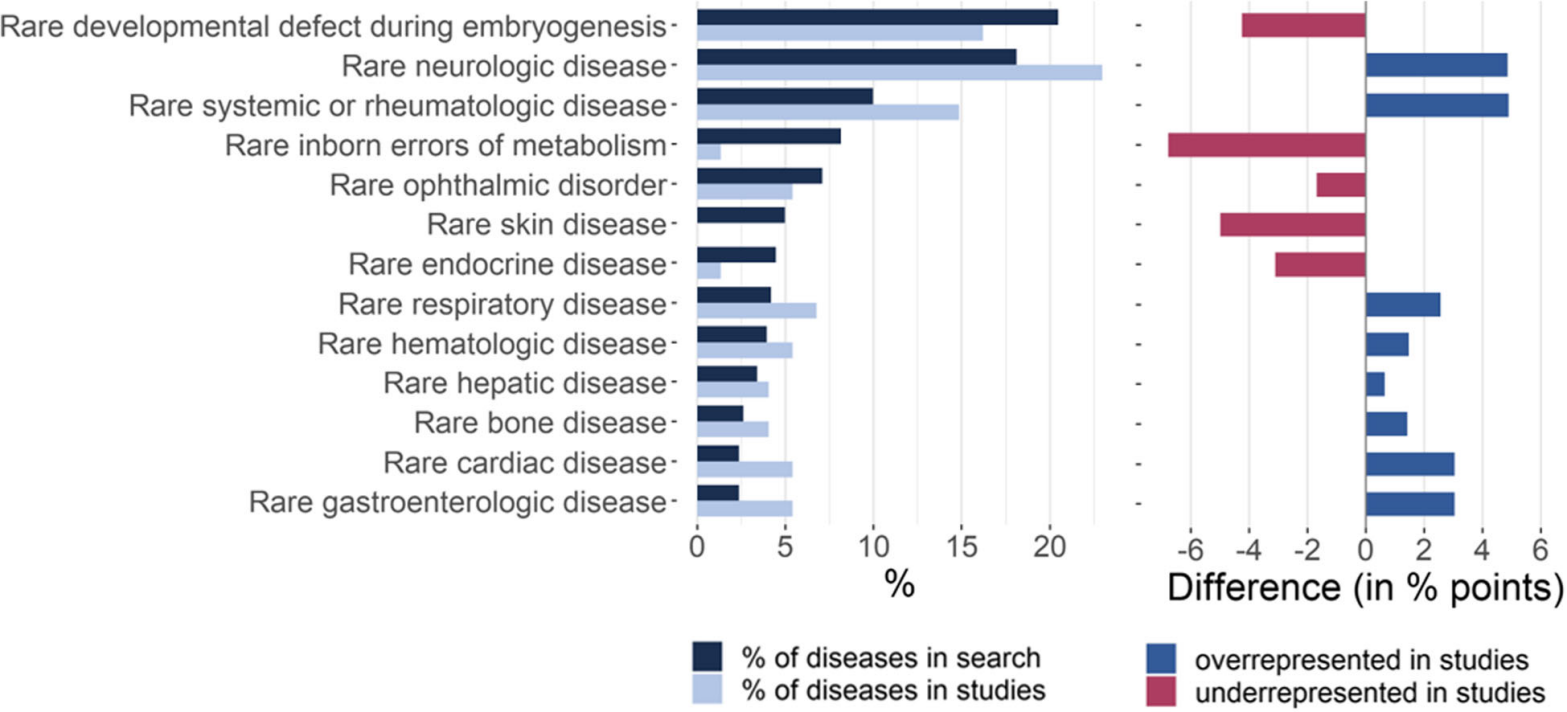
Distribution across disease groups: The distribution of the 381 diseases included in the literature search is shown in comparison with the distribution of the 74 diseases investigated in the studies (left; disease groups smaller than 3% are not shown); differences between the percentages show disease groups that are over- or underrepresented in the studies (right)

Comparing the distribution of disease groups investigated in the studies with the expected distribution (i.e., the “baseline” distribution of the diseases included in the literature search) revealed some groups that appeared to be overrepresented in the studies: Rare neurologic diseases, rare systemic or rheumatologic diseases, rare respiratory diseases, rare cardiac diseases and rare gastroenterologic diseases were investigated more frequently than to be expected (to a lesser extent, also rare hematologic and rare bone diseases). Conversely, other disease groups appeared to be underrepresented: Rare developmental defects during embryogenesis, rare inborn errors of metabolism, rare skin diseases and rare endocrine diseases were investigated less frequently than to be expected from their distribution in the search string (Fig. 3). For example, there were no studies on rare skin diseases, although the Orphanet list used in the literature search included 19 rare skin disorders (5.0%).

**Fig. 4:**
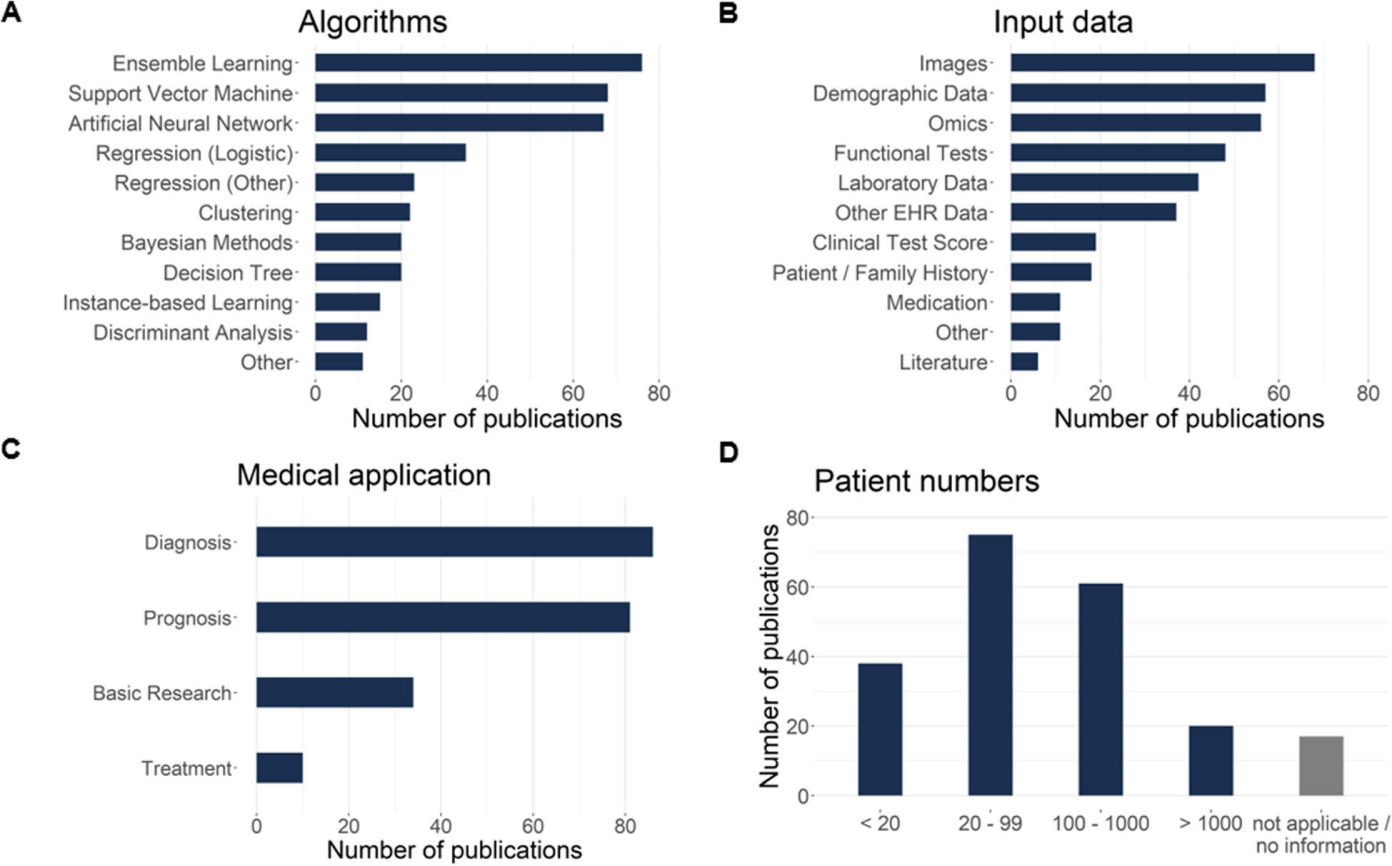
Types of algorithms used in the studies (**a**); input data (**b**); medical application (**c**); number of patients (**d**). Studies using more than one type of algorithm or input data are listed in more than one category

The algorithms most frequently used in the studies were ensemble methods (*n* = 76, 36.0%), support vector machines (*n* = 68, 32.2%) and artificial neural networks (*n* = 67, 31.8%) (Fig. 4a). Most frequent input data used by the algorithms were images (*n* = 68, 32.2%), demographic data (*n* = 57, 27.0%) and omics data (*n* = 56, 26.5%) (Fig. 4b). Most studies used machine learning for diagnosis (*n* = 86, 40.8%) or prognosis (*n* = 81, 38.4%), whereas studies aiming to improve treatment were relatively scarce (*n* = 10, 4.7%) (Fig. 4c). The number of patients investigated in the studies ranged from a few cases to several thousands, with studies typically using data from 20 to 99 patients (*n* = 75, 35.5%) (Fig. 4d). Twenty-five studies (11.8%) used an external data set to validate their algorithm; 5 studies (2.4%) validated their algorithm against a medical expert.

## Discussion

In this scoping review, we explored the scientific literature about machine learning methods used in the context of rare diseases. In particular, we investigated in which rare diseases and disease groups machine learning was typically applied, which types of algorithms and input data were used and which medical applications were studied.

Considering the large number of known rare diseases, the number of diseases investigated in the machine learning studies identified in this review was relatively small. The majority of diseases was in the highest prevalence class (1–5 / 10,000 patients), despite the search string including more diseases in the lower prevalence class (1–9 / 100,000 patients). Moreover, a large proportion of studies investigated a few relatively “common” or well-known rare diseases, such as amyotrophic lateral sclerosis, lupus or cystic fibrosis. This shows that the pattern that applies to rare diseases in general also seems to apply within the group of rare diseases: Diseases with a comparatively high prevalence are investigated more frequently whereas diseases with a lower prevalence are “orphans” that receive less attention. (However, note that our literature search might have missed some studies about diseases with a very low prevalence of 1–9 / 1,000,000 or lower because these diseases were not explicitly included in the search string and could only be identified via the generic rare disease search strings.)

Our review also revealed some rare disease groups that were investigated more frequently than to be expected from their occurrence in the search string. For example, the number of studies investigating rare neurologic diseases, rare systemic or rheumatologic diseases, rare respiratory diseases, rare cardiac diseases and rare gastroenterologic diseases was higher than to be expected. This observation can partly be explained by the prevalence of the diseases within a disease groups, i.e. disease groups containing more diseases with higher prevalence being investigated more frequently in the studies (as described in the previous paragraph). However, there were also disease groups – for example neurologic diseases – that were overrepresented in the studies, despite containing more diseases with a lower prevalence. For these disease groups the availability of data may play an important role: Many of the overrepresented disease groups work with imaging data (e.g., MRI data for neurologic diseases), which lend themselves particularly well for their use with machine learning. Some disease groups may also appear more frequently because they are part of large medical disciplines (e.g., neurology, rheumatology, cardiology etc.), which are not limited to rare conditions, and which can therefore draw on a large pool of existing research and methods.

There were also disease groups underrepresented in the studies. Most interestingly, our review did not identify any machine learning studies about rare skin diseases. This is surprising, as the diagnosis of skin conditions is often cited as one of the prime examples of successful machine learning applications in medicine [13, 27]. Developing machine learning applications for the diagnosis of rare skin conditions could therefore be a highly promising field of research. Similarly, rare inborn errors of metabolism and rare developmental defects during embryogenesis were also underrepresented in the studies and could possibly benefit from machine learning research – in particular because they constitute two of the most common groups of rare diseases.

Investigating typical algorithms, we identified ensemble methods, support vector machines and artificial neural networks as the algorithms most frequently used in the studies. Again, the choice of algorithms in the studies could be partly due to the data available to the algorithms. Images were identified as the most common type of input data, and the algorithms typically used in the studies (e.g., artificial neural networks) work well with this type of data. Moreover, image data (such as MRI, PET or CET) are acquired in large quantities in medical practice and can be processed in a relatively standardized way, thus providing a good data source for machine learning. The barrier of applying machine learning to other types of data, such as unstructured text data in medical records, is higher because these data are often not standardized and therefore more difficult to process. This highlights the importance of international health IT standards and medical terminologies that can improve interoperability and that can help to make medical data more accessible to machine learning [28]. In the context of rare diseases, standard vocabularies such as SNOMED CT [29], the Orphanet rare disease nomenclature [30] or the Human Phenotype Ontology (HPO) [31, 32] could particularly facilitate data interoperability.

Only a relatively small proportion of the studies in this review tested their algorithms on an external validation data set or validated performance against human experts. However, to facilitate translation of machine learning methods into clinical practice, appropriate validation is crucial. Machine learning studies should therefore aim to evaluate their performance on external data so that their potential for real-world application can be more easily assessed (of course, this applies to machine learning in general, not only in the context of rare diseases). Note that our review did not evaluate the performance of the machine learning algorithms, since the studies identified in this scoping review were too heterogeneous to perform meaningful comparisons across studies. To investigate algorithm performance, more specific systematic literature reviews and meta-analyses are needed (for example, focusing on specific diseases, input data or outcome variables).

Most studies identified in this review focused on diagnosis and prognosis of rare diseases. Considering that these are typical applications of machine learning (i.e., classification and prediction), this is not surprising. However, machine learning can also play an important role in improving the treatment of rare diseases, and future studies could focus more on this aspect, for example by using machine learning to accelerate drug development [33].

As to be expected in the context of rare diseases, the number of patients included in the studies was relatively small. Comparable reviews investigating machine learning in more common diseases, for example in diabetes mellitus [34], cancer [35] or coronary artery disease [36], have access to larger pools of patient data. This is important, as the performance of machine learning algorithms largely depends on the amount of data available for training the algorithms. The lack of sufficient training data could also explain why rare diseases with a higher prevalence were investigated more often than lower prevalence diseases. It is therefore important to further promote cross-institutional and international collaboration to create data sets sufficiently large for machine learning research.

## Conclusion

Advances in machine learning can significantly improve diagnosis, treatment and prognosis of rare disease patients. This scoping review explored more than 200 scientific studies from a 10-year time period to assess the use of machine learning in rare diseases. Our findings provide a broad overview for researchers and healthcare professionals, which can guide future research and inspire more specific systematic literature reviews and meta-analyses. Our findings also point to promising areas of future research that are underrepresented in current studies (e.g., using machine learning to diagnose rare skin conditions).

## Supplementary information

**Additional file 1.** Articles_included: List of articles included in the review and extracted data; Articles_all: All articles identified by the literature search and reasons for exclusion (if excluded); Search_string: Search strings used for the systematic literature search.

## Data Availability

The data generated and analyzed in this study are included in the supplementary information file.

## Abbreviations

AI: Artificial intelligence
ALS: Amyotrophic lateral sclerosis
CORD-MI: Collaboration on Rare Diseases
CT: Computed tomography
ECG: Electrocardiography
EEG: Electroencephalography
EJP RD: European Joint Programme on Rare Diseases
EMG: Electromyography
ERNs: European Reference Networks
HPO: Human Phenotype Ontology
HSCT: Hematopoietic stem cell transplantation
MRI: Magnetic resonance imaging
NCBI: National Center for Biotechnology Information
PET: Positron emission tomography
PRISMA: Preferred reporting items for systematic reviews and meta-analyses
PRISMA-ScR: PRISMA extension for scoping reviews (PRISMA-ScR)
UDN: Undiagnosed Diseases Network

## References

[1] European Commission. https://ec.europa.eu/info/research-and-innovation/research-area/health-research-and-innovation/rare-diseases_en. Accessed 16 Apr 2020.

[2] EURORDIS. https://www.eurordis.org/about-rare-diseases. Accessed 16 Apr 2020.

[3] Wakap SN, Lambert DM, Olry A, Rodwell C, Gueydan C, Lanneau V, et al. Estimating cumulative point prevalence of rare diseases: analysis of the Orphanet database. Eur J Hum Genet. 2020;28:165–173.. 10.1038/s41431-019-0508-0 PMC6974615 31527858

[4] Shire, Rare Disease Impact Report. https://globalgenes.org/wp-content/uploads/2013/04/ShireReport-1.pdf. Accessed 16 Apr 2020.

[5] Orphanet. http://www.orpha.net. Accessed 16 Apr 2020.

[6] Thompson R, Johnston L, Taruscio D, Monaco L, Béroud C, Gut IG (2014). RD-connect: an integrated platform connecting databases, registries, biobanks and clinical bioinformatics for rare disease research. J Gen Intern Med.

[7] European Reference Networks. https://ec.europa.eu/health/ern_en. Accessed 16 Apr 2020.

[8] European Joint Programme on Rare Diseases. https://www.ejprarediseases.org. Accessed 16 Apr 2020.

[9] Ramoni RB, Mulvihill JJ, Adams DR, Allard P, Ashley EA, Bernstein JA (2017). The undiagnosed diseases network: accelerating discovery about health and disease. Am J Hum Genet.

[10] Collaboration on Rare Diseases (CORD-MI). https://www.medizininformatik-initiative.de/en/CORD. Accessed 16 Apr 2020.

[11] Rajkomar A, Dean J, Kohane I (2019). Machine learning in medicine. N Engl J Med.

[12] Topol E (2019). Deep medicine: how artificial intelligence can make healthcare human again.

[13] Esteva A, Kuprel B, Novoa RA, Ko J, Swetter SM, Blau HM (2017). Dermatologist-level classification of skin cancer with deep neural networks. Nature.

[14] Liang H, Tsui BY, Ni H, Valentim CCS, Baxter SL, Liu G (2019). Evaluation and accurate diagnoses of pediatric diseases using artificial intelligence. Nat Med.

[15] Gulshan V, Peng L, Coram M, Stumpe MC, Wu D, Narayanaswamy A (2016). Development and validation of a deep learning algorithm for detection of diabetic retinopathy in retinal fundus photographs. JAMA.

[16] Ronicke S, Hirsch MC, Türk E, Larionov K, Tientcheu D, Wagner AD (2019). Can a decision support system accelerate rare disease diagnosis? Evaluating the potential impact of Ada DX in a retrospective study. Orphanet J Rare Dis.

[17] Gurovich Y, Hanani Y, Bar O, Nadav G, Fleischer N, Gelbman D (2019). Identifying facial phenotypes of genetic disorders using deep learning. Nat Med.

[18] Brasil S, Pascoal C, Francisco R, Dos Reis Ferreira V, Videira PA, Valadão AG. Artificial Intelligence (AI) in Rare Diseases: Is the Future Brighter? Genes. 2019;10:978. 10.3390/genes10120978 PMC6947640 31783696

[19] Arksey H, O’Malley L (2005). Scoping studies: towards a methodological framework. Int J Soc Res Methodol.

[20] Levac D, Colquhoun H, O’Brien KK (2010). Scoping studies: advancing the methodology. Implement Sci.

[21] Peters MDJ, Godfrey CM, Khalil H, McInerney P, Parker D, Soares CB (2015). Guidance for conducting systematic scoping reviews. Int J Evid Based Healthc.

[22] Munn Z, Peters MDJ, Stern C, Tufanaru C, McArthur A, Aromataris E (2018). Systematic review or scoping review? Guidance for authors when choosing between a systematic or scoping review approach. BMC Med Res Methodol.

[23] Tricco AC, Lillie E, Zarin W, O’Brien KK, Colquhoun H, Levac D (2018). PRISMA extension for scoping reviews (PRISMA-ScR): checklist and explanation. Ann Intern Med.

[24] Orphadata, Rare diseases and classifications. http://www.orphadata.org/cgi-bin/rare_free.html. Accessed 16 Apr 2020.

[25] R Core Team. R: a language and environment for statistical computing. https://www.R-project.org. Accessed 16 Apr 2020.

[26] Wickham H, Averick M, Bryan J, Chang W, McGowan LD, François R (2019). Welcome to the tidyverse. J Open Source Softw.

[27] Brinker TJ, Hekler A, Utikal JS, Grabe N, Schadendorf D, Klode J (2018). Skin Cancer classification using convolutional neural networks: systematic review. J Med Internet Res.

[28] Lehne M, Sass J, Essenwanger A, Schepers J, Thun S (2019). Why digital medicine depends on interoperability. NPJ Digit Med.

[29] SNOMED International. http://www.snomed.org. Accessed 16 Apr 2020.

[30] Rath A, Olry A, Dhombres F, Brandt MM, Urbero B, Ayme S (2012). Representation of rare diseases in health information systems: the orphanet approach to serve a wide range of end users. Hum Mutat.

[31] Robinson PN, Köhler S, Bauer S, Seelow D, Horn D, Mundlos S (2008). The human phenotype ontology: a tool for annotating and analyzing human hereditary disease. Am J Hum Genet.

[32] Groza T, Köhler S, Moldenhauer D, Vasilevsky N, Baynam G, Zemojtel T (2015). The human phenotype ontology: semantic unification of common and rare disease. Am J Hum Genet.

[33] Réda C, Kaufmann E, Delahaye-Duriez A (2020). Machine learning applications in drug development. Comput Struct Biotechnol J.

[34] Kavakiotis I, Tsave O, Salifoglou A, Maglaveras N, Vlahavas I, Chouvarda I (2017). Machine learning and data mining methods in diabetes research. Comput Struct Biotechnol J.

[35] Kourou K, Exarchos TP, Exarchos KP, Karamouzis MV, Fotiadis DI (2015). Machine learning applications in cancer prognosis and prediction. Comput Struct Biotechnol J.

[36] Alizadehsani R, Roshanzamir M, Abdar M, Beykikhoshk A, Khosravi A, Panahiazar M (2019). A database for using machine learning and data mining techniques for coronary artery disease diagnosis. Sci Data.

